# Risk factors of caesarean deliveries in urban–rural areas of Bangladesh

**DOI:** 10.3389/frph.2023.1101400

**Published:** 2023-02-15

**Authors:** Faruq Abdulla, Md. Moyazzem Hossain, Md. Mahabubur Rahman, Md. Siddikur Rahman, Azizur Rahman

**Affiliations:** ^1^ Department of Applied Health and Nutrition, RTM Al-Kabir Technical University, Sylhet, Bangladesh; ^2^ Department of Statistics, Jahangirnagar University, Savar, Bangladesh; ^3^ School of Mathematics, Statistics & Physics, Newcastle University, Newcastle Upon Tyne, United Kingdom; ^4^ Department of Statistics, Faculty of Sciences, Islamic University, Kushtia, Bangladesh; ^5^ Department of Statistics, Begum Rokeya University, Rangpur, Bangladesh; ^6^ School of Computing, Mathematics and Engineering, Charles Sturt University, Wagga Wagga, NSW, Australia

**Keywords:** C-section, prevalence, BDHS, logistic regression, Bangladesh

## Abstract

**Background and aims:**

The key interest of this research is to identify the causes of the ongoing increasing trends in caesarean section or C-section (CS) deliveries in both urban and rural areas of Bangladesh.

**Methods:**

This study analyzed all Bangladesh Demographic and Health Survey (BDHS) datasets through Chi-square and z tests and the multivariable logistic regression model.

**Results:**

CS deliveries were found to be more prevalent in urban than in rural areas of Bangladesh. Mothers above 19 years, above 16 years at first birth, overweight mothers, those with higher educational levels, those who received more than one antenatal care (ANC) visit, fathers having secondary/higher education degrees and employed as workers or in business, and mothers living in wealthy households in the cities of Dhaka, Khulna, Mymensingh, Rajshahi, and Rangpur divisions had a significantly higher likelihood of CS deliveries in urban areas. Contrastingly, mothers with ages between 20 and 39 years, above 20 years at first birth, normal weight/overweight mothers, those with primary to higher level of education, those in the business profession, fathers who also received primary to higher education, mothers who received more than one ANC visit, and those living in wealthy households in Dhaka, Khulna, Mymensingh, Rajshahi, and Rangpur divisions were more likely to have CS deliveries in rural areas. The 45–49 age group mothers had a five times higher likelihood of CS deliveries [odds ratio (OR): 5.39] in urban areas than in rural areas. Wealthy mothers were more likely to be CS-delivered in urban (OR: 4.84) than in rural areas (OR: 3.67).

**Conclusion:**

The findings reveal a gradual upward alarming trend in CS deliveries with an unequal contribution of significant determinants in urban and rural areas of Bangladesh. Therefore, integrated community-level awareness programs are an urgent need in accordance with the findings on the risks of CS and the benefits of vaginal deliveries in this country.

## Introduction

A caesarean section can be a life-saving intervention when medically indicated, but it can also trigger many short-term and long-term adverse health complications for both mother and baby (1–4). CS should be executed only when there is a medical necessity and the standard rate of CS is maintained at approximately 10%–15% (3). However, CS deliveries have been increasing noticeably worldwide during the past two decades and are also placing a high clinical and economic burden on healthcare systems (5–8). The growth in CS deliveries has been revealed to have adverse implications for the health of infants as well as mothers and increased the costs of deliveries (9–11). In comparison with vaginal or natural births, CS deliveries performed on non-medical hints in low-resource settings are linked to higher maternal hazards (7), lengthier postpartum recovery (12), higher rates of rehospitalization (13), prolonged hospital stays (14), greater risk of maternal morbidity (15), and difficulties in subsequent pregnancies (16).

A study showed that the global average CS rate increased by 12.4% in the period between 1990 and 2014, with the maximum average annual rate of increase happening in Asia (2). In Bangladesh, the rate of CS deliveries increased from 4% to 23% between the years 2004 and 2014 (4) and one-third of such deliveries occurred in 2018 (17). Moreover, the percentage of CS deliveries in Bangladesh is significantly higher than that in neighboring countries like Pakistan (14%), India (14%), and Nepal (4%) (4). There are several factors triggering the increment of CS. In most developing countries, social and educational improvements and demographic changes are the main cause for delayed pregnancies among mothers until they reach the end of their fertile lives (18). Studies have found a higher likelihood of CS among shorter mothers (19) and younger mothers with a small pelvis (20). Mothers having better socioeconomic status (21), belonging to upper social classes, highly educated ones, and living in urban and metropolitan areas are more likely to prefer CS (22–28). The high prevalence of national CS is predominantly due to the excessive rate of CS triggered by the richest population living in urban areas in South Asia as well as other low-middle-income countries (29, 30). Generally, CS is observed among mothers whose baby sizes are either smaller than average or very large, have a higher education level, and the place of delivery is a private medical institution (31). Several variables such as age, education, wealth, and the number of antenatal visits were found to be significantly positively associated with CS deliveries among low-risk mothers in India (32).

Various studies conducted in South Asian countries, including Bangladesh, have pointed out to increased concerns about the higher rate of CS and predict that the national increase in the CS rate could be partially motivated by private health facilities that are mainly driven by profit maximization (30, 33, 34). A few studies have found that some physicians conduct CS for economic gains and time management without any medical justification (35). Hospitals’ financial and organizational structures (36, 37) also influence critical decisions. The increase in monetary gains through CS encourages many health providers to choose CS (38, 39). Given the above discussion, it is therefore essential to identify the trends and the most important predictors and their influence on CS deliveries. Some studies on these already exist in the literature, but there is a significant research gap on the issue of urban–rural divide in CS deliveries in Bangladesh. Therefore, this study aims to explore the trends in CS deliveries and their determinants among Bangladeshi mothers at their reproductive age in urban–rural areas using Bangladesh Demographic and Health Survey (BDHS) data.

## Material and methods

### Data source

This study considered all published BDHS datasets (i.e., 1993/94, 1996/97, 1999/00, 2003/04, 2007, 2011, 2014, and 2017/18) to examine the trends in CS delivery rates. However, an assessment of the association of CS deliveries with different socioeconomic and demographic characteristics and subsequent analysis is based on the most recent secondary data collected from BDHS-2017/18. The sampling frame of this survey was the list of enumeration areas (EAs) of the 2011 Population and Housing Census of the People's Republic of Bangladesh. The primary sampling unit of the survey was an EA. The survey used a two-stage stratified sampling technique. In the first stage, 675 EAs were chosen, with 227 and 448 EAs from urban and rural areas. However, data could not be collected from three EAs because of the occurrence of natural disaster. These clusters were in Dhaka (one urban cluster), Rajshahi (one rural cluster), and Rangpur (one rural cluster). In the second stage, a systematic sample of 30 households was selected from each EA. A total of 20,250 residential households were selected in four phases. Among the 20,376 ever-married women aged 15–49 years and eligible for interviews, 20,127 were interviewed, yielding a response rate of approximately 99%. The detailed sampling procedure is available in the report of BDHS-2017/18 (17).

### Outcome Variable

The outcome variable in the present study was a dichotomous variable, CS delivery, (i) No or (ii) Yes. This variable was measured by asking a question to the participants, “Did you ever give birth by CS?”.

### Covariates

In this study, the mother's age in years (15–19, 20–24, 25–29, 30–34, 35–39, 40–44, and 45–49), age at 1st birth (≤16, 17–20, 21–24, and 25 or more years), body mass index (BMI) (underweight, normal, overweight/obesity), educational level (no education, primary, secondary, higher), occupation (not working, worker, business, service), the father’s education level (no education, primary, secondary, higher), the father's occupation (not working, worker, business, service), birth order (1, 2–3, 4 or more), number of antenatal visits during pregnancy (no visits, 1–4, 5–8, and 9 or more visits), religion (Muslim, non-Muslim), place of residence, wealth index (moderately poor, poorest, middle class, moderately rich, richest), and division (Barisal, Chittagong, Dhaka, Khulna, Mymensingh, Rajshahi, Rangpur, and Sylhet) were all considered covariates.

### Statistical analysis

In this study, initially, bivariate analysis (Chi-square test) was performed to determine significant associations between mode of birth (caesarean vs. non-caesarean) and select sociodemographic factors. These associated variables were considered independent variables for the logistic regression model (unadjusted and adjusted), which was implemented to find the most influential factors for CS delivery. The logistic regression model can be expressed as

$$\mathrm{Pr}\;\left(Y_{i}=1\right)=\frac{\exp\;\left(X_{i}\beta \right)}{1+\exp\;\left(X_{i}\beta \right)}$$

where *Y*_*i*_ is a binary variable that takes a value of “1” if the respondent received CS delivery and “0” otherwise; *X*_*i*_ is a vector of independent variables and β is a vector of unknown parameters that consist of the intercept parameter and the regression parameter associated with a set of covariates used in the study. The fitted form of the model can be defined as

$$\ln\;\left[\frac{{\hat{P}}_{i}}{1-{\hat{P}}_{i}}\right]={\hat{\beta }}_{0}+{\hat{\beta }}_{1}X_{1}+\cdots +{\hat{\beta }}_{k}X_{k},$$

where ${\hat{\beta }}_{p}=(p=0,1,2\ldots ,k)$ represents the estimated regression coefficient of the *p*-th independent variable in the study.

## Results

**Figure 1:**
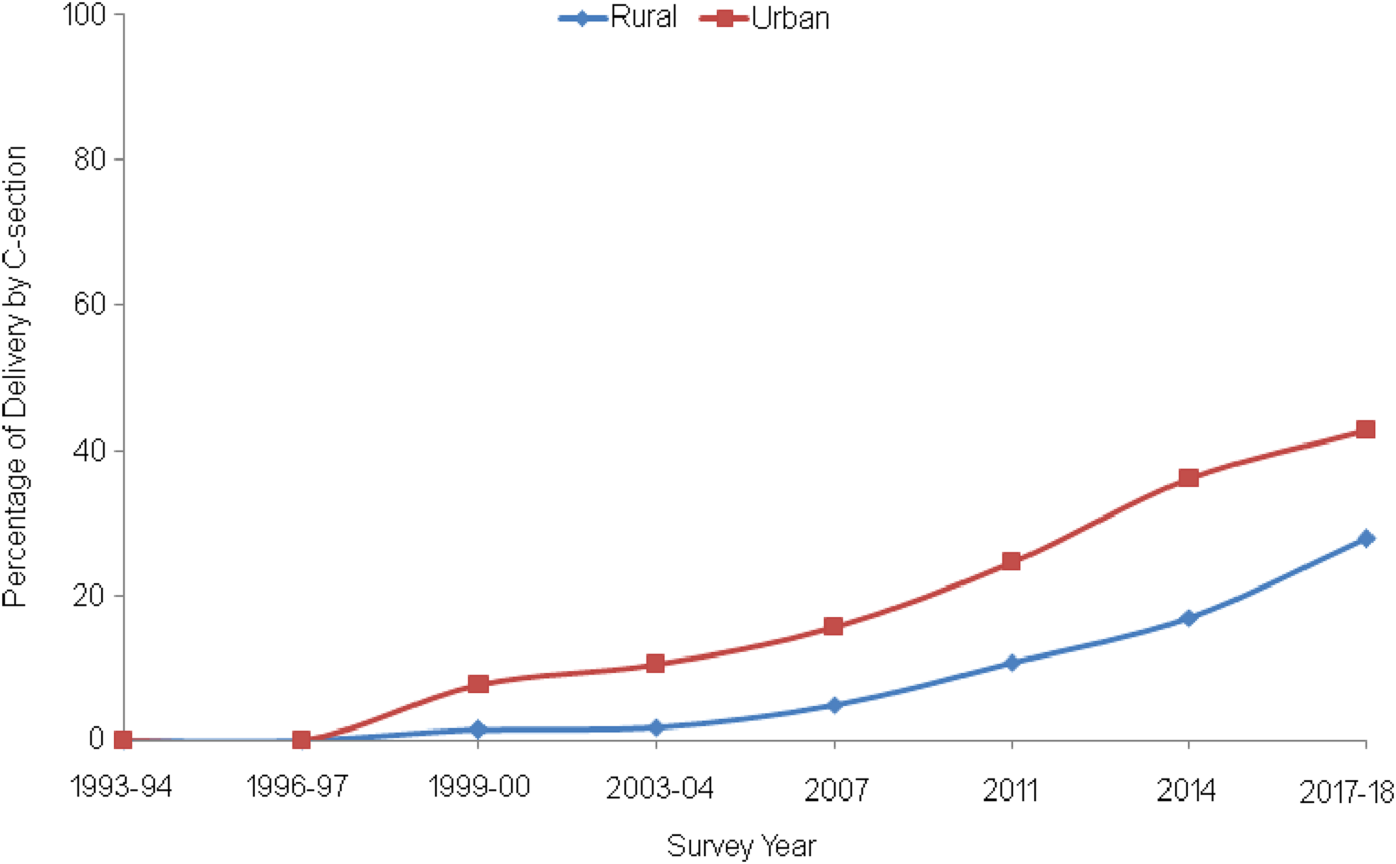
Trends in CS deliveries by type of residence from survey years 1993/94 to 2017/18.

Figure 1 illustrates the percentage of delivery by CS in Bangladesh's urban and rural areas between the survey years 1993 and 2018. CS deliveries in urban areas are more common than in rural areas for all survey years. It is interesting to observe that no CS deliveries are reported in both areas between 1993 and 1996. However, after 1996, the percentage of such deliveries increases gradually from 0% in 1996 to approximately 43% in 2018 in urban areas. On the other hand, it is found that CS deliveries in rural areas slowly increase from 0% in 1996 to approximately 5% in 2007. Then, it increases sharply from approximately 5% in 2007 to approximately 28% in 2018. The overall trend is upward in both urban and rural areas of Bangladesh, but there is a significant gap between the prevalence of CS deliveries between the respondents’ places of residence.

**Figure 2:**
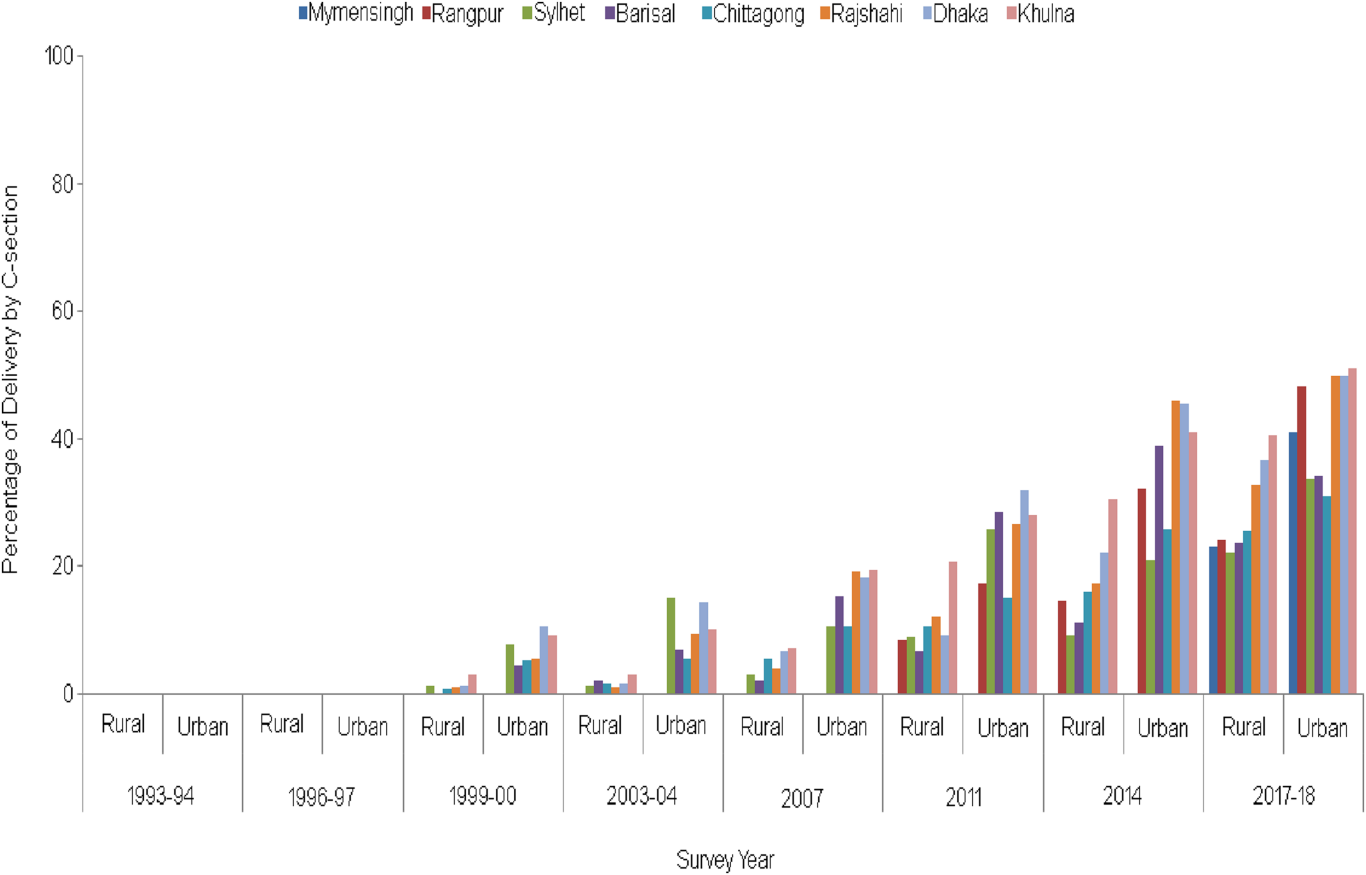
Trends in CS deliveries by division and type of residence from 1993/94 to 2017/18 (note: before 1995, there were only five divisions in Bangladesh, and subsequently, Sylhet, Rangpur, and Mymensingh divisions were added in 1995, 2010, and 2015, respectively).

The percentage of delivery by CS based on ever-married women aged 15–49 years, separated by location, namely, rural and urban regions, is presented in Figure 2. We observe that CS deliveries are always higher in urban areas than in rural areas in the country’s geospatial divisions. CS deliveries are the most popular choice in the rural areas of Khulna division for all survey years, followed by Dhaka, Rajshahi, and Chittagong for the last two survey years.

CS deliveries are widespread in the urban areas of Khulna, Dhaka, and Rajshahi divisions. In contrast, such deliveries are less common in Barisal division's rural and urban areas, followed by the Chittagong division. However, in the Sylhet division, CS was popular initially, but after 2014, the prevalence is less observable. Overall, for the entire survey period, an increasing trend in the percentage of CS is observed in all parts of the country’s urban and rural regions.

**Figure 3:**
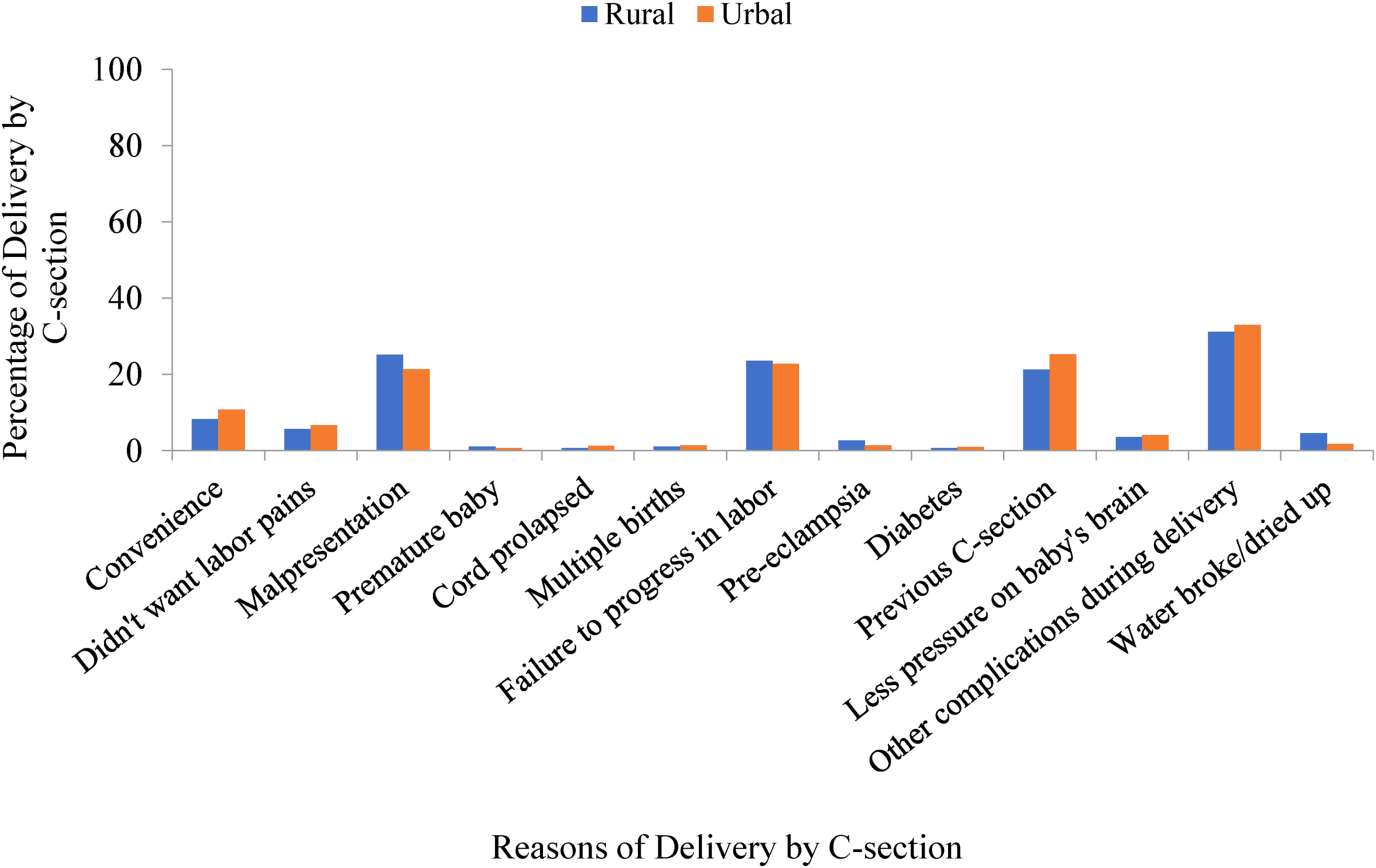
Distribution of CS deliveries by factors in the survey year 2017/18.

The bar chart portrayed in Figure 3, which takes into account the BDHS-2017/18 data, shows the percentage of CS deliveries grouped according to the different reasons for such deliveries in urban and rural areas. Reasons such as convenience, unwilling to bear labor pains, cord prolapse, multiple births, diabetes, previous CS, less pressure on the baby's brain, and other complications during delivery were found to be higher for urban areas than for rural areas. In contrast, reasons such as malpresentation, premature baby, failure to progress in labor, preeclampsia, and broken/dried up water were found to be more in rural areas. Overall, the main reasons for CS were malpresentation (approximately 21% in urban areas and 25% in rural areas), failure to progress in labor ( approximately 23% in urban areas and 24% in rural areas), and previous CS (approximately 25% in urban areas and 21% in rural areas) (Figure 3).

The associations between the prevalence of CS and other sociodemographic, socioeconomic, geographic, and antenatal care (ANC)-related variables were investigated by using the chi-square test, and the results are given in Table 1. Interestingly, it is observed that all the considered covariates influence the prevalence of CS in both urban and rural areas of Bangladesh. Although the mother's age is associated with CS prevalence with a *p*-value < 0.001 in urban areas, the association is also significant in rural areas with a *p*-value of 0.085. On the other hand, religion shows a significant association in only rural areas with a *p*-value of 0.001. Because the difference between the proportions of CS in urban–rural areas can be explicitly visualized, the significance of this difference can also be justified by using the z-test in terms of every covariate considered in this study. The results of the test statistic, along with the *p*-value, are presented in Table 1. The results demonstrate that the CS delivery rate in urban areas is significantly different from that in rural areas in terms of all covariates. It is fascinating that the observed *p*-values are less than 0.001 in all levels of the considered variables, except in the wealth index, which is categorized as a middle-class parameter, which denotes that the father does not engage in any work. However, the difference between the two variables of urban and rural areas is also significant, in that there is a 10% level of significance, since the *p*-values of the wealth index (middle) denoting that the father does not work are 0.002 and 0.075, respectively (Table 1).

**Table 1:** Association between mode of birth and socioeconomic and demographic factors of Bangladeshi mothers aged 15–49 years, BDHS-2017/18.

Factors	Labels	Delivery by caesarean section									
		Urban				Rural				Test of equality of proportion of CS delivery in urban and rural	
		No	Yes	Chi-square	*P*-value	No	Yes	Chi-square	*P*-value	*z*-statistic	*P*-value
**Mother's age (**y**ear)**	15–19	197	90	25.75	<0.001	433	168	11.11	0.085	1.05	<0.001
	20–24	350	242			859	373			4.48	<0.001
	25–29	247	218			654	212			8.33	<0.001
	30–34	147	130			361	137			5.45	<0.001
	35–39	42	48			121	39			4.61	<0.001
	40–44	15	9			18	6			0.93	<0.001
	45–49	2	2			4	0			1.63	<0.001
Age of mother at first birth **(**y**ear)**	≤16	293	109	156.7	<0.001	764	194	102.84	<0.001	2.78	<0.001
	17–20	533	323			1,329	486			5.75	<0.001
	21–24	143	190			297	181			5.40	<0.001
	25 or more	31	117			60	74			4.27	<0.001
BMI	Underweight	167	74	108.35	<0.001	467	105	97.42	<0.001	3.88	<0.001
	Normal	620	336			1,644	575			5.28	<0.001
	Overweight/obesity	213	329			339	255			5.99	<0.001
Mother's highest educational level	No education	82	26	227.19	<0.001	199	24	251.87	<0.001	3.17	<0.001
	Primary	338	93			844	158			2.65	<0.001
	Secondary	449	315			1,183	506			5.48	<0.001
	Higher	131	305			224	247			5.40	<0.001
Mother's occupation	Not working	661	536	55.91	<0.001	1,287	628	91.51	<0.001	6.72	<0.001
	Worker	258	94			1,039	232			3.50	<0.001
	Business	10	11			17	10			1.06	<0.001
	Service	71	98			107	65			3.73	<0.001
Husband's education level	No education	135	43	214.97	<0.001	464	72	255.78	<0.001	3.37	<0.001
	Primary	384	133			981	250			2.50	<0.001
	Secondary	332	239			771	348			4.39	<0.001
	Higher	149	324			234	265			4.91	<0.001
Husband's occupation	Not working	6	6	45.97	<0.001	16	10	58.66	<0.001	0.67	0.075
	Worker	377	166			1,315	367			4.15	<0.001
	Business	244	223			441	201			5.56	<0.001
	Service	373	344			678	357			5.66	<0.001
Birth order number	1	374	347	40	<0.001	811	447	100.42	<0.001	5.50	<0.001
	2–3	494	356			1,234	435			8.09	<0.001
	4 or more	132	36			405	53			3.13	<0.001
Number of ANC visits during pregnancy	0	80	7	168.3	<0.001	308	17	186.87	<0.001	1.00	<0.001
	1–4	589	272			1,515	487			4.04	<0.001
	5–8	270	345			543	357			6.30	<0.001
	9 or more	61	115			84	74			3.41	<0.001
Wealth index combined	Poorest	178	21	263.67	<0.001	801	122	315.27	<0.001	1.02	<0.001
	Moderately poor	106	38			714	193			1.38	<0.001
	Middle	173	74			466	213			0.41	0.002
	Moderately rich	298	159			342	221			1.47	<0.001
	Richest	245	447			127	186			1.57	<0.001
Religion	Muslim	918	678	0.002	0.967	2,266	832	10.719	<0.001	10.86	<0.001
	Non-Muslim	82	61			184	103			1.36	<0.001
Division	Barisal	109	54	49.24	<0.001	290	91	63.72	<0.001	2.23	<0.001
	Chittagong	185	83			428	148			1.60	<0.001
	Dhaka	209	206			205	119			3.51	<0.001
	Khulna	97	100			196	131			2.39	<0.001
	Mymensingh	95	66			352	106			4.35	<0.001
	Rajshahi	77	79			259	127			3.85	<0.001
	Rangpur	86	81			306	97			5.73	<0.001
	Sylhet	142	70			414	116			3.16	<0.001
**Overall**	1,000	739			2,450	935			10.75	<0.001

BDHS, Bangladesh Demographic and Health Survey; CS, caesarean section; BMI, body mass index; ANC, antenatal care.

**Figure 4:**
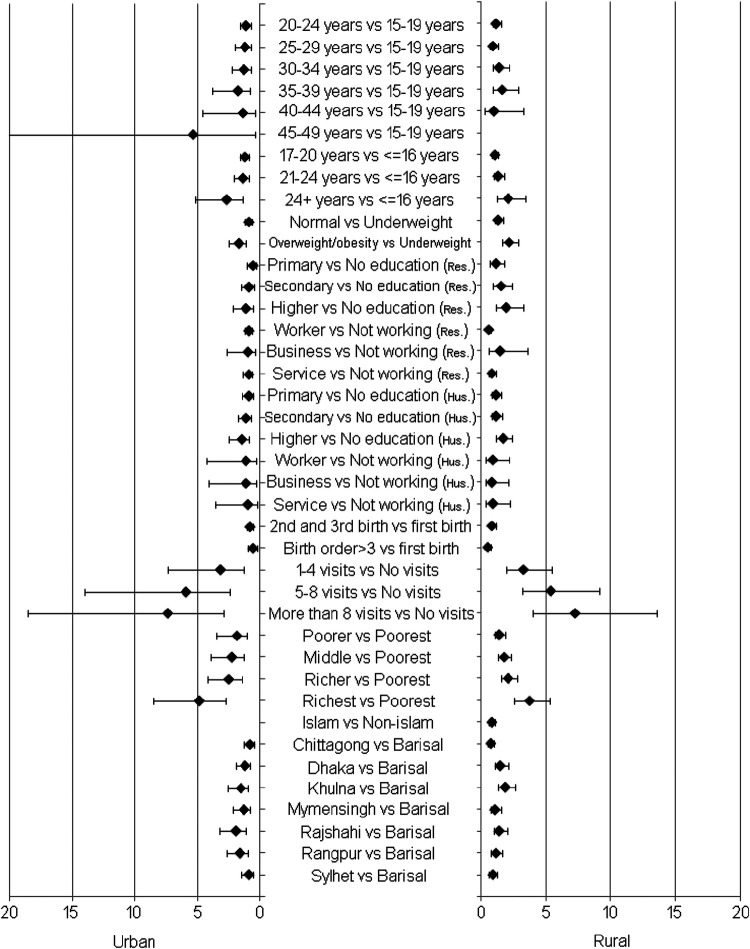
Forest plot for odds ratios with 95% confidence intervals corresponding to the considered factor's labels in urban versus rural areas.

Then, the multivariable logistic regression model with CS as the dependent variable and the identified significant covariates as the independent variables was used to measure the impact of the covariates on CS. The results are presented in Table 2 and Figure 4. The geographical region (Khulna and Rajshahi division) showed a significant association with CS. Mothers who lived in the urban areas of Rajshahi [odds ratio (OR): 1.9] and rural areas of Khulna (OR: 1.8) were significantly more likely to undergo CS compared with those in Barisal. The odds of CS were found higher among better-educated mothers than their less-educated counterparts in both urban and rural areas. Better-educated mothers in rural areas had significantly higher odds (OR: 1.8) of having a CS delivery. Mothers who belonged to higher-wealth quintiles had more odds of undergoing CS in both urban and rural areas, for example, moderately poor (urban: 1.8, rural: 1.3), middle class (urban: 2.2, rural: 1.7), moderately rich (urban: 2.4, rural: 2.0), and richest (urban: 4.8, rural: 3.6). Overweight/obese mothers (BMI > 30) had higher odds of having a CS delivery compared with their underweight counterparts in both urban (OR: 1.6) and rural (OR: 2.0) areas. The odds of obese mothers having a CS delivery, in both urban (OR: 1.6) and rural (OR: 2.0) areas, were significantly higher than those who were underweight (Figure 4).

**Table 2:** Summary of the logistic regression of CS delivery on the sociodemographic, economic, geographic, and ANC-related factors.

Factors	Labels	**Urban**				**Rural**			
		B	SE	Sig.	OR	B	SE	Sig.	OR
**Mother's age (**y**ear)**	Ref: 15–19
	20–24	0.10	0.19	0.60	1.11	0.12	0.14	0.41	1.12
	25–29	0.21	0.25	0.41	1.23	−0.14	0.18	0.45	0.87
	30–34	0.25	0.29	0.38	1.28	0.30	0.22	0.17	1.36
	35–39	0.56	0.39	0.15	1.76	0.44	0.30	0.15	1.56
	40–44	0.30	0.63	0.63	1.36	−0.07	0.63	0.92	0.94
	45–49	1.69	1.28	0.19	5.39	−19.94	19,140.80	1.00	0.00
Age of mother at fir**st birth (**y**ear)**	Ref: ≤16
	17–20	0.16	0.16	0.31	1.18	−0.01	0.11	0.93	0.99
	21–24	0.33	0.22	0.13	1.39	0.24	0.16	0.14	1.27
	25 or more	0.99	0.33	0.00	2.68	0.70	0.26	0.01	2.02
BMI	Ref: Underweight
	Normal	−0.13	0.18	0.46	0.88	0.25	0.13	0.05	1.29
	Overweight/obesity	0.53	0.20	0.01	1.69	0.74	0.16	0.00	2.09
Mother's highest educational level	Ref: No education
	Primary	−0.55	0.29	0.06	0.58	0.07	0.25	0.79	1.07
	Secondary	−0.15	0.28	0.60	0.86	0.38	0.25	0.13	1.46
	Higher	0.13	0.33	0.69	1.14	0.63	0.28	0.02	1.88
Mother's occupation	Ref: Not working
	Worker	−0.16	0.16	0.33	0.85	−0.61	0.10	0.00	0.55
	Business	0.00	0.50	1.00	1.00	0.35	0.47	0.46	1.42
	Service	−0.09	0.20	0.65	0.91	−0.28	0.19	0.14	0.75
Husband's education level	Ref: No education
	Primary	−0.10	0.23	0.65	0.90	0.11	0.16	0.48	1.12
	Secondary	0.12	0.24	0.60	1.13	0.13	0.17	0.44	1.14
	Higher	0.37	0.27	0.17	1.45	0.48	0.20	0.02	1.62
Husband's occupation	Ref: Not working
	Worker	0.14	0.67	0.83	1.15	−0.16	0.47	0.74	0.85
	Business	0.11	0.67	0.87	1.11	−0.20	0.47	0.67	0.82
	Service	−0.04	0.67	0.96	0.97	−0.13	0.47	0.79	0.88
Birth order number	Ref: 1
	2–3	−0.25	0.17	0.14	0.78	−0.19	0.13	0.14	0.83
	4 or more	−0.64	0.33	0.05	0.53	−0.80	0.24	0.00	0.45
Number of ANC visits during pregnancy	Ref: 0
	1–4	1.14	0.44	0.01	3.13	1.17	0.26	0.00	3.21
	5–8	1.77	0.44	0.00	5.88	1.67	0.27	0.00	5.33
	9 or more	2.00	0.47	0.00	7.40	1.98	0.32	0.00	7.25
Wealth index combined	Ref: Poorest
	Moderately poor	0.64	0.32	0.05	1.89	0.31	0.14	0.02	1.36
	Middle	0.81	0.29	0.01	2.26	0.54	0.15	0.00	1.71
	Moderately rich	0.91	0.27	0.00	2.49	0.70	0.16	0.00	2.00
	Richest	1.58	0.29	0.00	4.84	1.30	0.19	0.00	3.67
Religion	Ref: Non-Muslim
	Muslim	−0.29	0.15	0.06	0.75	−0.18	0.12	0.14	0.83
Division	Ref: Barisal
	Chittagong	−0.26	0.25	0.31	0.77	−0.37	0.18	0.04	0.69
	Dhaka	0.21	0.23	0.38	1.23	0.36	0.19	0.06	1.43
	Khulna	0.45	0.26	0.08	1.57	0.59	0.19	0.00	1.81
	Mymensingh	0.27	0.27	0.31	1.31	0.03	0.18	0.87	1.03
	Rajshahi	0.65	0.27	0.02	1.91	0.31	0.18	0.09	1.36
	Rangpur	0.47	0.27	0.09	1.59	0.07	0.19	0.73	1.07
	Sylhet	−0.11	0.27	0.67	0.89	−0.18	0.19	0.33	0.83
Constant		−3.28	0.90	0.00	0.04	−2.93	0.64	0.00	0.05

CS, caesarean section; ANC, antenatal care.

Mothers who received a higher number of antenatal care visits during pregnancy also had higher odds of undergoing CS delivery in both urban and rural areas, for example, 1–4 visits (urban: 3.2, rural: 3.2), 1–4 visits (urban: 5.8, rural: 5.3), and 8 or more (urban: 7.3, rural: 7.2) compared with 0 visits. Rural mothers who were employed had less odds of CS done on them (OR = 0.5) than those who were not. The likelihood of CS deliveries was higher among mothers at first birth in the age group of (24 + years) in urban (OR: 2.6) and rural areas (OR: 2.0) compared with the ≤16 year age group. The possibility of CS decreased with a higher birth order in both urban and rural areas; for example, a birth order of more than 3 in urban (OR = 0.5) and rural areas (OR = 0.4) was lower than that of first birth. The mother's age, religion, and the father’s occupation were not significantly associated with CS deliveries in both areas (Figure 4).

## Discussion

One of the key findings of this study is that the CS delivery rate is continuously increasing over time in urban and rural residents. In a study by Khan et al., it is reported that the prevalence rate of CS has been increasing in Bangladesh over the last few decades (40). However, after a period of 15 years, it is now approximately 11 times that of the first reported rate and nearly three times that of the WHO's recommended ideal rate of 10%–15% in both urban and rural areas of Bangladesh (3). Based on the data up to 2015, the WHO declared that CS rates of more than 10% were not associated with reductions in maternal and new-born mortality rates at the population level (41). The increased rate of CS delivery was associated with different health problems such as an increased risk of postpartum antibiotic use, maternal morbidity and mortality, fetal and neonatal morbidity, placenta accreta, reduced fetal growth, preterm delivery, pelvic pain, adverse reproductive effects, and many more (41). A more than 10% CS rate did not facilitate the provision of health improvement measures for mothers or newborns (42). Therefore, if CS delivery prevalence continues to increase in its current pace, it is highly likely that CS will exercise more harmful impacts at the population level (40). Given the geographical structure of Bangladesh, which is in the form of divisions, CS rates are found to be high in Khulna, followed by Dhaka, Rajshahi, Rangpur, Mymensingh, Chittagong, Barisal, and Sylhet in that order. Interestingly, the pattern of CS delivery practices is similar in Khulna and Dhaka divisions, while Chittagong and Barisal divisions closely follow. Similar findings were reported in 2013 (43).

Nevertheless, CS deliveries are increasing in both areas, but in urban areas, the popular appeal of such deliveries is more than two times that in rural areas over the past decade, and it was much higher in the previous decade. These findings indicate that a large number of urban mothers prefer CS compared with their rural counterparts. Many studies have identified urbanization as a significant contributing factor to CS practices in several countries (27, 30, 42, 44–51). The prevalence of CS has been increasing in urban areas over several decades in low- and middle-income countries in Asia, Africa, and Latin America (27, 42, 47–51). A retrospective study was conducted to analyze data from demographic and health surveys, and it reported a rising CS trend in Pakistan’s urban areas compared with the rural areas (52). The CS rate has continuously increased over the years in Vietnam's urban area, which is similar to the trend observed in low- to middle-income countries like Bangladesh (53). However, the urban–rural divide is visible in every division in Bangladesh. The differences in CS delivery in urban and rural areas are multifactorial and complex in nature (40). Nonetheless, it is evident from previous studies that this urban–rural divide reflects the presence of different socioeconomic, demographic, and healthcare factors such as higher income and education level, easy accessibility to healthcare facilities, and easy availability of government, private, and non-government medical facilities for the antenatal care of pregnant mothers (43, 53, 54). In one study, it is mentioned that cultural, educational, and economic differences across areas might be the foremost reasons for region-wise variations in CS (55).

Another finding shows that “other complications during delivery (31.9%)” is the leading indication for CS, followed by malpresentation (23.4%), failure to progress in labor (23.2%), previous CS (22.9%), convenience (9.3%), and unwillingness to bear labor pain (6%). A study conducted in the Thakurgaon district of Bangladesh reported that the most typical indications for CS were previous CS (29.4%), fetal distress (15.7%), cephalopelvic disc proportion (10.2%), prolonged obstructed labor (8.3%), and post-term dates (7.0%) (46). Another study conducted in the International Centre for Diarrhoeal Disease Research, Bangladesh (ICDDR, B) service area in Bangladesh found that common indications for CS were absence of maternal complications (24.9%), absolute maternal indications (24.7%), failure to progress (16.5%), and no clear medical indication (12.5%) in a total of 401 CS deliveries (56). Fetal distress, preeclampsia, and cervical dystocia were identified as the most common CS indicators in Bangladesh’s urban areas (57). Moreover, fetal distress, previous CS, breech presentation, and slow progress in labor were the most familiar indicators for CS in Nepal (58). The WHO estimated that about one-third of the total CS deliveries were done without medical symptoms described as “unnecessary” (45).

These findings also indicate that the pregnant mother's age, age at first birth, BMI, education, occupation of the mother and her partner, birth order, ANC visits, wealth index, and geographic region (division) are the significant leading factors of CS deliveries in both urban and rural areas. Interestingly, religion is a significant leading factor of CS in rural areas. The factors mentioned above were also identified as potential significant predictors of CS deliveries in many previous studies (4, 25, 31, 32, 40, 53–55). Moreover, these results also indicate that the identified significant leading factors do not uniformly influence CS delivery prevalence in urban and rural areas. It has been reported that the cultural, educational, and economic differences across areas are the main reasons for the regional variations in CS deliveries (55).

Moreover, this study measured the risk of pregnant mothers’ CS deliveries from a different viewpoint of the significant leading factors. It found that older mothers were more likely to experience different complications during pregnancy and delivery (23, 25, 59–61), which increased the likelihood of CS delivery. These findings are similar to our results from urban areas; however, we observe different results in rural areas. As with previous findings (54, 62), this study also found a higher likelihood of CS delivery among mothers who were obese and aged 24 or more at first birth, and the chances of CS decreased with a higher birth order. We found that mothers preferred CS because of the increased risk of complications in other deliveries. Urban mothers aged 24 or more at first birth had a greater possibility of undergoing CS than their rural counterparts, while obese mothers in rural areas had a higher possibility of undergoing CS than urban mothers.

Our findings indicate that mothers with higher education are more likely to undergo CS. This finding is similar to previous studies’ results (40, 54, 55). A better-educated rural mother has a higher likelihood of undergoing CS than her urban-educated counterpart, but the exact reason for this is not clear from this study. Furthermore, this study found that CS delivery and birth order were inversely related in both urban and rural areas. A similar result was identified in many previous studies (43, 54, 63). In addition, mothers living in households with higher socioeconomic status and those who received higher ANC visits during pregnancy were more likely to opt for CS. Our findings are consistent with those of earlier studies that also explored the influence of maternal education, wealth status, and ANC visits on the use of maternity care services, especially CS delivery (4, 54, 64). Higher-educated and wealthiest mothers plumped for CS deliveries because they had a higher ability to pay (62) to receive specialized care (4). The influence of both factors (ANC visits and wealth index) on CS was higher in urban than in rural areas. In a study, data collected from 80 demographic and health surveys from 26 countries in Southern Asia or sub-Saharan Africa were analyzed, and it was found that the wealthiest urban mothers were more likely to undergo CS than the wealthiest rural mothers (30).

Furthermore, mothers whose husbands are also better educated are more likely to accept CS than those whose husbands are less educated, with the likelihood being slightly greater in urban areas. Working mothers are the least likely to opt for CS compared to non-working mothers in both areas. This may be due to the fact that working mothers have less chances of receiving ANC services because of time constraints (65). Likewise, Muslim mothers are less likely to undergo CS deliveries compared with non-Muslim mothers in urban and rural areas. This finding is consistent with that of a previous study, which reported that Muslim mothers have less opportunities to avail of ANC services due to their religious beliefs and the restrictions imposed by their husbands on the breach of privacy (66). Mothers whose husbands are employed in rural areas are less likely to undergo CS, but those whose husbands are professionals or businessmen in urban areas are more likely to undergo CS. Compared with mothers in Barisal division, those in Dhaka, Khulna, Mymensingh, Rajshahi, and Rangpur divisions have a greater likelihood of having CS, while mothers in Chittagong and Sylhet divisions are less likely to have CS in both urban and rural areas. Also, urban mothers are more likely to undergo CS than rural mothers in Dhaka, Khulna, Mymensingh, Rajshahi, and Rangpur. These findings are consistent with those of previous studies (43, 40). However, these findings are quite alarming for a low- and middle-income country like Bangladesh (4, 54, 64), because the situation can place a heavy financial burden on the healthcare system and family economic status in the country (4). Furthermore, due to the lack of a robust quality control system, there is a possibility that hospitals/clinics in Bangladesh may aim for profit maximization, and similar settings in other countries may produce similar results (4).

The main strength of this study is its novelty. Second, this study was carried out with country representative data. Third, methodological robustness increased the acceptability of the findings. The main limitation of this study is that it was conducted with cross-sectional data, and therefore, causal inferences could not be derived. Second, there may be other contributing factors to CS delivery that were not considered in this study.

### Implications for practice and/or policy

The increasing trends in CS deliveries in urban areas is approximately two- to sixfold higher than in rural areas between the years 1993 and 1994 and between 2017 and 2018, and this indicates an undesirable situation in the context of Bangladeshi healthcare. Many factors have already been identified previously for the increasing number of CS deliveries in the country. However, this study focused on the urban–rural variations in CS deliveries. It is suggested that policymakers should design community-based maternal healthcare programs to reduce the prevalence rates of CS and also the economic burden placed on both urban and rural settings in light of the findings of this research.

## Conclusion

This study has been conducted in the urban and rural areas of Bangladesh with the principal aim of examining the CS delivery pattern over time and to identify its causes and influential factors. The trend analysis showed that CS delivery prevalence has increased over the past two decades in both areas with apparent differences. Moreover, the increasing trends and the urban–rural divide have also manifested in Bangladesh's geographic divisions. Furthermore, extraneous complications during delivery have been the leading indications for CS, followed by malpresentation, failure to progress in labor, previous CS, convenience, and unwillingness to bear labor pain in both urban and rural areas, however, with a slight difference. However, among these causative factors, CS delivery due to only malpresentation is higher in rural areas than in urban areas, while the prevalence of the remaining factors is higher in urban areas. The analysis confirmed that CS delivery is significantly influenced by the pregnant mother's age, age at first birth, BMI, education, occupation of both mother and husband, birth order, ANC visits, wealth index, and geographic division in both urban and rural areas; religion is an important covariate in rural areas. These findings reveal that the aforementioned influential factors of CS vary in terms of the resident settings (urban or rural) of pregnant mothers.

This study found that urban mothers were more likely to undergo CS deliveries than their rural counterparts. This was mainly the case of those who were more than 19 years old, overweight, had higher education levels, received more than one ANC visit, were aged greater than 16 years at first birth and whose husbands were secondary or higher educated and professionals or businessmen, and lived in wealthy households in Dhaka, Khulna, Mymensingh, Rajshahi, and Rangpur divisions. On the other hand, mothers with age between 20 and 39 years, age at first birth >20 years, normal or overweight, received primary to higher education, business professionals, whose husbands were also primary to higher educated, received more than one ANC visit, and belonged to wealthy families in Dhaka, Khulna, Mymensingh, Rajshahi, and Rangpur divisions were more likely to undergo CS deliveries in rural areas. However, the increasing practice of CS delivery is posing a threat to traditional and normal deliveries, and for a low- and middle-income country like Bangladesh, CS can place a heavy financial burden on the healthcare system and family economic status. Therefore, the government of Bangladesh needs to act fast by developing new policies and regulations to make sure that CS is carried out only when it is medically appropriate to do so and not for the sole purpose of deriving financial benefits.

## Data Availability

Publicly available datasets were analyzed in this study. These can be found in https://dhsprogram.com.
